# Comparing Intramedullary Nails Versus Dynamic Hip Screws in the Treatment of Intertrochanteric Hip Fractures on Post-operative Rehabilitation Outcomes:A Systematic Review Protocol

**DOI:** 10.1177/21514593221144180

**Published:** 2022-12-05

**Authors:** Steve Papp, Chantal Backman, Lauren Konikoff, Peter Tanuseputro, Anne Harley, Soha Shah, Randa Berdusco, Marie-Cécile Domecq, Stéphane Poitras, Veronique French-Merkley, Paul E. Beaulé

**Affiliations:** 1 The Ottawa Hospital, Ottawa, ON, Canada; 2 Bruyère Research Institute, Ottawa, ON, Canada; 3 27337 Ottawa Hospital Research Institute, Ottawa, ON, Canada; 4 University of Ottawa, Ottawa, ON, Canada; 5 Bruyère Continuing Care, Ottawa, ON, Canada

**Keywords:** intramedullary nails, dynamic hip screws, intertrochanteric hip fractures, post-operative rehabilitation outcomes

## Abstract

**Background:** Intertrochanteric hip fractures are treated by fixation with either an intramedullary (IM) Nail or Dynamic Hip Screw (DHS). It is unknown whether one surgery type has better post-operative rehabilitative outcomes for the hip fracture population. This systematic review aims to compare post-operative rehabilitation outcomes of intertrochanteric hip fractures treated via IM Nails versus DHS. **Methods:** We will conduct a systematic review following the Cochrane Handbook and the Preferred Reporting Items for Systematic Reviews and Meta-Analyses (PRISMA) format. A search strategy will be developed, and the following databases will be searched: MEDLINE, EMBASE, Cochrane Library, and Web of Science. Two reviewers will perform a two-step screening process and data extraction of included studies. Any disagreement will be resolved with a discussion or a third reviewer. Risk of bias and the quality of the studies will also be assessed. A narrative synthesis will be used for the data analysis. **Conclusion:** This systematic review will provide evidence for orthopaedic surgeons and rehabilitation clinicians to further improve patient rehabilitation outcomes post-hip fracture surgery.

## Background

Intertrochanteric hip fractures are treated by fixation with either an intramedullary (IM) Nail or Dynamic Hip Screw (DHS). In the literature, both intramedullary (IM) Nails and Dynamic Hip Screws (DHS) have shown favourable results.^1-3^

The decision of whether to use IM Nail vs DHS is not consistently applied in practice. In principle, IM Nail is a stronger construct than the DHS fixation.^4^ Therefore, many authors have suggested that DHS fixation can be used for most *stable* intertrochanteric fractures^5^ but more *unstable* patterns should be treated by IM Nail.^6^ Interestingly, several studies demonstrate that even the treatment of unstable fracture patterns using DHS has equal success rates of proper healing and therefore IM Nail fixation is not superior in stable or unstable fracture patterns.^7,8^ Since both techniques have shown favourable results and the DHS is less costly, it might make sense that the less expensive implant be used more frequently. Despite this concept, orthopaedic surgeons tend to favor the use of IM Nail and their use has grown significantly over the past 20 years.^2,9^

Although it is important to consider the “success” of surgery based on a fracture healing, there are also other considerations including operating room time, blood loss, length of stay, patient-reported outcomes, hospital discharge location, and rehabilitation success. We know that some hip fracture patients have difficulty returning to their pre-fracture level of function and multiple factors likely play a role.^10^ When comparing the DHS and IM Nail, Bohl and colleagues found that patients being treated with an IM Nail were discharged on average, one day earlier than those who received the DHS as a treatment type.^1^ Bienkowski and colleagues also found that patients treated with IM Nail fixation were more likely to recover the ability to ambulate well six months post-surgery compared with DHS patients (71% vs 33%, respectively).^11^

However, most studies to date have focused primarily on health care utilization related outcomes^1,2,9,11,12^ and to our knowledge, less research has specifically examined whether IM Nails or DHS fixation leads to better post-operative rehabilitation outcomes. Thus, we aim to synthesize the existing literature that compares post-operative rehabilitation outcomes of intertrochanteric hip fractures treated via IM Nails versus DHS. We will also describe the outcomes that are currently evaluated in the literature and identify gaps for future research.

## Methods

### Research Design and Methodology

We will conduct a systematic review following the Preferred Reporting Items for Systematic Reviews and Meta-Analyses (PRISMA) format.^13^ This protocol was prepared in accordance with the Preferred Reporting Items for Systematic Review and Meta-Analysis Protocols (PRISMA-P) statement.^13^ This review is also registered with PROSPERO (CRD42022364556).

### Eligibility Criteria

The inclusion and exclusion criteria are as follows: ***Population:*** intertrochanteric hip fractures. ***Interventions:*** intramedullary (IM) Nails. ***Comparison:*** Dynamic Hip Screws (DHS). ***Outcomes:*** rehabilitation (e.g, time to initiation of rehabilitation, functional outcomes, quality of life, rehabilitation length of stay, rehabilitation intensity/frequency, type of rehabilitation, discharge destination), health utilization (e.g., return to the emergency room, readmission to hospital), and survival outcomes. ***Study designs:*** randomized controlled trials and non-randomized experimental studies (e.g., cohort, case-control, controlled before-and-after, interrupted-time-series, and controlled trials not using full randomization), qualitative or mixed-methods primary studies.

### Search Strategy

The search strategy was developed by an information specialist and was peer-reviewed by another information specialist following the Peer Review of electronic search strategies (PRESS) guidelines.^14^ The following databases will be searched: MEDLINE, EMBASE, Cochrane, and Web of Science. The MEDLINE search strategy can be found in Appendix 1. All citations will be imported into Covidence,^15^ a systematic review management software, where duplicates will be removed.

### Screening

Two reviewers (LK, CB) will perform a two-step screening process. Specifically, the two reviewers will independently screen titles and abstracts (level 1 screening) according to the pre-determined eligibility criteria. For the level 2 screening, the two reviewers will independently screen the full texts. Any disagreement will be resolved by a discussion or by a third reviewer (SP). The reasons for exclusion will be noted using the Preferred Reporting Items for Systematic Reviews and Meta-Analyses (PRISMA) format.^13^ We will manually search the reference lists of all the included studies and relevant systematic reviews. Relevant best practice guidelines will also be hand searched.

### Data Extraction

Prior to starting the data extraction, we will pilot test our data extraction form in Microsoft Excel. Two reviewers (LK, CB) will independently extract the data from the eligible studies. This will include full reference, country, purpose, study design, type of participants, number of participants, theoretical approach, description of the intervention, data analysis, and study results/outcomes. Any disagreement will be discussed to reach an agreement.

### Risk of Bias Assessment

The risk of bias of each study will be assessed independently by two reviewers. We will use the Cochrane risk-of-bias tool for randomized trials (RoB 2)^16^ to assess the risk of bias in randomized trials and the Risk of Bias in non-randomized studies of interventions (ROBINS-I)^17^ to assess the risk of bias in non-randomized studies comparing two or more interventions. All conflicts will be resolved by a discussion or if needed, by a third reviewer (SP). We will not exclude any studies in our review based on their quality. We will present the studies’ weaknesses if it has an overall impact on the data synthesis.

### Quality of the Evidence

We will use the Grading of Recommendations Assessment, Development and Evaluation (GRADE) framework^18^ to evaluate the quality of the body of evidence from studies. The risk of bias across studies will be assessed through evaluation of the publication bias using a funnel plot. A funnel plot will be constructed by plotting the effect estimates reported in each study against their effect sizes. Publication bias will be assessed if the included studies have a heterogeneous sample size and their study design and methodology do not differ considerably.

### Data Synthesis

We will conduct a narrative data synthesis. Data will be grouped by settings, intervention type, outcome, and study design. All data tables will contain data on setting, intervention and control, study sample, patient characteristics, study design, outcome, and overall RoB. Where possible, a meta-analysis of results from randomized controlled studies and non-randomized studies will be carried out to estimate a summary measure of effect. Meta-analysis will be performed using Review Manager 5.^19^

## Conclusion

This systematic review will provide evidence for orthopaedic surgeons and rehabilitation clinicians to further improve patient rehabilitation outcomes post-hip fracture surgery. This research will also identify gaps and provide recommendations to inform further work by determining which approach may provide better functional recovery for intertrochanteric hip fracture patients.

## Appendix 1

### MEDLINE search strategy

Ovid MEDLINE(R) ALL <1946 to August 10, 2022>

1 exp Hip Fractures/27586
2 ((intertrochanteric adj3 fracture*) or IFT).ti,ab,kw,kf. 4254
3 ((Trochanteric or subtrochanteric) adj3 fracture*).ti,ab,kw,kf. 3047
4 ((Hip? or femur? or femoral) adj3 fracture*).ti,ab,kw,kf. 46029
5 or/1-4 54311
6 Bone Screws/25269
7 ((Dynamic adj2 Hip adj2 Screw*) or dhs).ti,ab,kw,kf. 4522
8 ((bone or hip?) adj3 (pin? or screw*)).ti,ab,kw,kf. 5657
9 or/6-8 31561
10 Fracture Fixation, Intramedullary/11133
11 Bone Nails/12165
12 ((Intramedullary adj3 nail*) or IM or PFN).ti,ab,kw,kf. 57658
13 ((fixation or nail* or rod?) adj3 (intramedullary or interlock* or inter-lock* or kuntscher?)).ti,ab,kw,kf. 11054
14 (Proximal adj3 (nail* or intramedullary)).ti,ab,kw,kf. 1554
15 or/10-14 71443
16 5 and 9 and 15 1393
17 limit 16 to (english or french) 1121
